# *Acinetobacter baumannii* Infection in Transfusion Dependent Thalassemia Patients with Sepsis

**DOI:** 10.1155/2017/2351037

**Published:** 2017-05-17

**Authors:** Muzaheed, Faisal Mousa Alzahrani, Saeed Sattar Shaikh

**Affiliations:** Department of Clinical Laboratory Science, College of Applied Medical Sciences, University of Dammam, Dammam, Saudi Arabia

## Abstract

**Purpose:**

To identify the* Acinetobacter baumannii* infection among transfusion dependent thalassemia patients.

**Methods:**

A quantitative approach was employed to assess* Acinetobacter baumannii* infection in transfusion dependent thalassemia patients. Samples were collected from 916 patients, which have shown bacterial growth on MacConkey and blood agar culture media.* A. baumannii* strains were identified by microbiological methods and Gram's staining. API 20 E kit (Biomerieux, USA) was used for final identification.

**Results:**

From 916 cultured blood specimens, 107 (11.6%) showed growth of* A. baumannii*. Serum ferritin in thalassemic patients without bacterial infections was 3849.5 ± 1513.5 *µ*g/L versus 6413.5 ± 2103.9 *µ*g/L in those with bacterial infections (*p* = 0.0001).* Acinetobacter baumannii* infected patients have shown higher serum ferritin levels (*p* = 0.0001). Serum ferritin in thalassemic patients was 3849.5 ± 1513.5 *µ*g/L versus 6413.5 ± 2103.9 *µ*g/L in those with bacterial infections (*p* = 0.0001).* Acinetobacter baumannii* infected patients showed high serum ferritin levels (*p* = 0.0001). The clinical symptoms have been found with* A. baumannii* +ve with a mean and standard deviation of 47 (5.1%) and* A. baumannii* −ve with mean and standard deviation of 60 (6.5%).

**Conclusion:**

Isolation of asymptomatic* A. baumannii* from the thalassemia patients shows an alarming situation of bacterial infections. A continuous surveillance of transfusion dependent thalassemia patients is recommended for bacterial sepsis.

## 1. Introduction

*Acinetobacter baumannii* belongs to the family of gram-negative bacteria [1]. The bacteria has been mostly found in the clinical samples, particularly related to the nosocomial infections [2]. Nosocomial infections are mainly received through sinks, shower units, infusion pumps, resuscitation apparatuses, and fomites, such as pillows, mattresses, and sinks. Hospital water systems have often been recognized as a source of nosocomial infection principally among the patients, who are immunocompromised in critical care units [3]. Furthermore, it is also identified as the etiological factor for the blood infections in patients in critical condition [4–6]. The hospital acquired infections are a global concern that affect a significant number of patients during treatments. More specifically, intensive care units (ICUs) tend to be contaminated with the pathogens that conveniently result in cross transmission. As the bacteria can survive and grow on dry surfaces, the contagion can also be passed by the hands of the healthcare professional and even environment [6, 7].

*Acinetobacter baumannii*, as a typical opportunistic pathogen, expresses a myriad of factors, which affect their pathogenicity in humans. These elements have the ability to settle and persist clinging to solid surfaces. Also, they have the ability to be extracted from the surrounding nutrients, in particularity to iron. The bacteria work by adherence to the epithelial cells and their subsequent death by apoptosis that is preceded by the production and secretion of enzymes. These products tend to be toxic in nature and are capable of infecting and damaging tissues. However, limited knowledge is presently known about the molecular and biochemical nature of most of these processes and factors, chiefly about the role of virulence and pathogenesis related to bacterial infections [2].

The bacterium* Acinetobacter baumannii* is presumed to have extensive association with the thalassemia patients due to the fact that these individuals require a regular blood transfusion for their management. This aspect makes them vulnerable to acquire infections and also develop iron overload in their body, which may provide a likely environment for the bacteria to flourish [8, 9]. The chances for the bacterial transmission are higher either from the contaminated equipment or the already infected blood [10–12]. The mortality, due to the septic shock as a result of blood infection, is one of the common observations in the cases of thalassemia [13]. As Pakistan comprises a large, transfusion dependent, population suffering from thalassemia, there is a huge gap for identifying the risk of bacterial contamination in these patients. Therefore, the study has aimed to identify the prevalence of* Acinetobacter baumannii* infection in transfusion dependent thalassemia patients, who have presented with sepsis, in the region of Sindh, Pakistan.

## 2. Materials and Methods

An observational study was carried out at* Diagnostic and Research Laboratory, Liaquat University of Medical and Health Sciences (LUMHS) Jamshoro/Hyderabad*, Sindh, Pakistan, from June 2015 to August 2015.

### 2.1. Inclusion Criteria

The patients, who presented with severe sepsis and required hospitalization management, were selected for the study from the outpatient department (OPD). The international criteria adopted for the diagnosis of sepsis were made on the clinical and radiological findings. Sepsis was confirmed by the culture and sensitivity of blood, urine, stool, pus, or other body fluid samples. It was implemented after the identification of the possible indications that may include inflammation or abscess, skin wounds, fever, neutropenia, shortness of breath, chest and/or abdominal pain, and backache that may relate to any recent trauma or prior injury [14].

The incidence of* Acinetobacter baumannii* was correlated to the clinical findings of iron overload, as initially the condition inclines to be asymptomatic; however, it may express dire outcomes of organ dysfunction and even failure when it persists [15]. The expression of symptoms has been indicated as positive (+ve) and the absence as negative (−ve). Serum ferritin level was used as a marker of the iron excess as it tends to increase during sepsis.

### 2.2. Samples

A total of 916 patients were recruited into the study during the observational period. The blood samples for the purpose of culture have been obtained after taking the signed consent from the participants. The blood sample was drawn by following the standardized approach for blood withdrawal. Specifically, the approach of venipuncture was used after the sanitization of skin through alcohol swab. A tourniquet was used during the process of blood withdrawal. A volume of 10 ml blood was taken from the patients through standardized blood withdrawal techniques. Moreover, the blood has been withdrawn once for the investigation.

### 2.3. Media, Reagents, and Kits

- MacConkey and blood agar (Oxoid Ltd., Cambridge, UK) medium were used for culture and sensitivity.
- Blood agar medium differentiates the hemolytic and nonhemolytic bacteria. Iso-Sensitest agar medium (Oxoid Ltd., Cambridge, UK) was used for the determination of resistance pattern against different antibiotics groups.
- Isolates were identified by API 20 E kit (Biomerieux, USA) [16].

### 2.4. Identification of Bacterial Isolates

Isolates have been identified via standard microbiological techniques, like colony morphology and Gram's staining [16]. Positivity of the cultures has been identified as the existence of infections; thus, such infections have been termed as symptomatic infections. Complete blood components were cultured to identify specified range of outcomes.

### 2.5. Data Analysis

The data was analyzed with SPSS 21.0 (IBM, incorporation, USA) statistical package. Continuous and categorical variables were analyzed by Student's *t*-test and Chi-square testing, respectively. The covariates were also analyzed through similar approach, which mainly included number of RBCs, patients' age, and exposure to chelation therapy. The difference of resistance levels of various drugs in ESBL producing strains versus non-ESBL producing strains was calculated by Fisher exact test. Data was analyzed at 95% confidence interval (*p* ≤ 0.05).

## 3. Results

**Figure 1:**
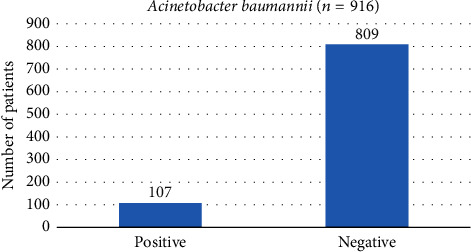
*Acinetobacter baumannii*.

Out of 916 cultured blood specimens, 107 (11.6%) specimens presented growth of* A. baumannii*. The study involved 66.81% of the males and 33.18% of the females. Majority of the thalassemic patients (86.5%) were chronic transfusion dependent, receiving two transfusions per week (Table 1). High serum ferritin levels were observed in the study population as shown in Table 1. Serum ferritin in thalassemic patients without bacterial infections was 3849.5 ± 1513.5 *µ*g/L and 6413.5 ± 2103.9 *µ*g/L in those with bacterial infections (*p* = 0.0001).* Acinetobacter baumannii* infected patients indicated high serum ferritin levels. None of the study participants had ever received iron chelation therapy. The clinical symptoms have been found with* A. baumannii* +ve with a mean and standard deviation of 47 (5.1%) and* A. baumannii* −ve with mean and standard deviation of 60 (6.5%) (Figure 1). The serum ferritin has also been found statistically significant (*p* = 0.0001) with and without bacterial infection.

## 4. Discussion

The present study has evaluated that there were high serum ferritin levels; and clinical symptoms were found with* A. baumannii* +ve. The serum ferritin has also been found statistically significant with and without bacterial infection. Iron overload is a usual clinical issue among patients with major *β*-thalassemia. A study by Akhlaghpoor et al. [17] assessed the occurrence of excess iron in some parts of the brain (basal ganglia, adenohypophysis, thalamus, and midbrain) among patients with *β*-thalassemia major and evaluated the relationship among serum ferritin and liver iron content. Liver iron content and serum ferritin might not be appropriate indicators of brain iron deposition in patients with major *β* thalassemia. It has been observed by Talsania et al. [18] that patients in the corporation hospitals had more frequency of blood transfusion as compared to the government hospital. Major thalassemia cases were advanced as compared to minor complications [18].* Acinetobacter baumannii* is found in the water, soil, and hospital environments in the first project reported in Sindh [19].* A. baumannii* is considered as a pathogen opportunistic and, therefore, it rarely causes infections. Generally, it affects the patients, who are hospitalized and underwent invasive procedures or are immunocompromised and use antineoplastic [11].

*A. baumannii* induced nosocomial infections include bacteremia, septicemia, endocarditis, pneumonia, wound infections, and urinary tract infections. It is a cause of bloodstream infections in the intensive care setting [20].* Acinetobacter baumannii* is probable to cause a variety of infections, including pneumonia, bacteremia, peritonitis, and urinary tract infection [2].* A. baumannii* organisms showed virulence factors for solid and dry surfaces to sequester iron from surrounding adhesion to epithelial surfaces, production of gelatinase and proteinases, skin colonization, and ability to form biofilms for colonization and survival [21]. During the last decade, treatment of these infections has become critical, depending on the emergence of multidrug-resistant strains, which has been associated with infection of hospital equipment (respirators, air-conditioning swimming, equipment for diagnostic imaging, etc.) [11]. The emergence of resistance to carbapenems has limited the treatment to the use of polymyxins, which is considered as the main therapeutic option. However, it has been observed that although the resistance to polymyxins is very rare in isolated* Acinetobacter*, clinical efficacy in the treatment of infections is not always satisfactory. It has been found that the biofilm synthesis due to these bacteria on a plastic medium was stimulated by iron deficiency in surrounding conditions imposed by the iron chelator 2,2′-dipyridyl (DIP) [19]. Hence, it was reported that the iron is needed for the biofilm mode of growth of* A. baumannii* [3].

Transfusion is an important therapy for thalassemia, but it contains risk including transfusion reactions, hemosiderosis, infections, and alloimmunization [22]. Risk factors, which predispose a patient for infection crab, are not distinct from other multiresistant microorganisms. Patients, who are multi-invaded and underwent surgical procedure, were most affected. Wang et al. [23] isolated bacteria flora from transfusion dependent thalassemia patients. Microorganisms included* K. pneumoniae, Acinetobacter baumannii, Streptococcus intermedius, Pseudomonas aeruginosa, Vibrio vulnificus, Yersinia enterocolitica*, and* Escherichia coli.* Wang et al. [23] have reported an incidence of severe sepsis of 1.60 infections/100 subjects annually.

The true incidence of bacterial infections in transfusion dependent thalassemic patients has been neglected in the area of clinical research. In the present study, 107 (11.68%) specimens showed growth of* A. baumannii* out of total 916 cultured blood specimens. The results are aligned with the study of Wang et al. [23]. However, the results of* A. baumannii *infection in transfusion dependent thalassemic patients remained unmatched as the present study is the first research to report from Sindh region. Regular transfusions might lead to more complicated situations like iron overload, if the patients are not treated appropriately. The transmission of HCV, HBV, and HCV is a main issue in the developing countries, where the standards of blood safety are not really high. In accordance with the findings of the study conducted by Ahmed Kiani et al. [24], Transfusion Transmitted Infections were distressingly high and HCV was observed as the leading TTI along with the presence of HIV in some cases.

Extensively drug resistant (XDR)* Acinetobacter baumannii* (Acb) might be the source of serious infections in censoriously ill patients. There is the only therapeutic option that is colistin that often remains helpful. Adding of rifampicin to colistin might be synergistic in vitro. The study assessed that the arrangement of rifampicin and colistin will condense the mortality of XDR Acb infections as compared to colistin alone. In serious XDR Acb infections, 30-day mortality is not reduced by the addition of rifampicin to colistin. The results highlighted that the rifampicin should not be regularly joint with colistin in clinical practice. The augmented rate of Acb annihilation with amalgamation treatment might still infer a clinical benefit [25].

When a potential threat is felt by the body, Fe (iron) gets transferred to ferritin to be contained, so that the injurious and damaging invader cannot get to the iron. Enough iron is obtained to make the red blood cells, but there is no surplus remained to nourish the injurious pathogens. A person with anemia will experience a modest decline depending on the underlying causes of disease. It will take place over time, observing the onset of inflammation because of the presence of disease or infection. The values of hemoglobin will reach a low and normal range of 9.5–10.5 g/dL and remain constant. Therefore, anemia of chronic disease can be recognized with a serum ferritin test [26].

As iron overload occurs in thalassemia patients because of regular blood transfusions particularly in those without iron chelating therapy, there are more chances of gram-negative bacterial infections. The presence of iron predisposes thalassemic patients to bacterial infections [3].* A. baumannii* sequesters iron by iron carriers and stores bound with host's proteins; and thus, it can combat decreased level of iron in blood [27].

It was found that* A. baumannii* shows good ability to sequester host's iron as it produces large quantities of siderophores for Fe (III) transport [28]. Keeping in mind the key role of iron in* Acinetobacter baumannii *pathogenicity [28, 29], iron chelator is considered as a nonantibiotic option to combat certain bacterial infection. As the transfusions are dependent on thalassemia patients, large quantities of iron are observed; therefore, it can be said that it is an ideal environment for the* A. baumannii* growth [2, 29].

## 5. Conclusion

The findings of* A. baumannii *from the thalassemia patients are supported by the previous studies as mentioned above. It is identified that iron overload is one of the risk factors for bacterial colonization. Isolation of* A. baumannii *from the thalassemia patients showed an alarming situation of bacterial infections. A continuous surveillance of transfusion dependent thalassemia patients is recommended for bacterial infections. Iron chelation therapy may alleviate the iron load in thalassemia patients.

## Figures and Tables

**Table 1:** Characteristics of study population (*n* = 916).

	Mean ± SD *N* (%)	*p* value
Age (years)	7.1 ± 2.5	
Male	612 (66.81%)	0.0001
Female	304 (33.18%)	
First transfusion age (years)	1.8 ± 1.3	
Years of transfusion (years)	6.3 ± 1.7	
Frequency of transfusions		
(i) 1 transfusion per week	123 (13.42%)	0.0001
(ii) 2 transfusions per week	793 (86.5%)	
Serum ferritin (*µ*g/L)	4989.3 ± 1978.3 *µ*g/L	
Serum ferritin (*µ*g/L) with bacterial infections	3849.5 ± 1513.5 *µ*g/L	0.0001
Serum ferritin (*µ*g/L) without bacterial infections	6413.5 ± 2103.9 *µ*g/L	
*Acinetobacter baumannii* infection		
(i) Positive	107 (11.68%)	0.0001
(ii) Negative	809 (88.31%)	
Clinical symptoms		
(i) *A. baumannii* +ve	47 (5.1%)	0.02
(ii) *A. baumannii* −ve	60 (6.5%)	
